# The Expression of Human Cytomegalovirus MicroRNA MiR-UL148D during Latent Infection in Primary Myeloid Cells Inhibits Activin A-triggered Secretion of IL-6

**DOI:** 10.1038/srep31205

**Published:** 2016-08-05

**Authors:** Betty Lau, Emma Poole, Benjamin Krishna, Immaculada Sellart, Mark R. Wills, Eain Murphy, John Sinclair

**Affiliations:** 1 Department of Medicine, University of Cambridge, Cambridge CB2 2QQ, United Kingdom; 2 Department of Molecular Genetics, Lerner Research Institute, Cleveland Clinic, Cleveland, Ohio 44195, United States of America

## Abstract

The successful establishment and maintenance of human cytomegalovirus (HCMV) latency is dependent on the expression of a subset of viral genes. Whilst the exact spectrum and functions of these genes are far from clear, inroads have been made for protein-coding genes. In contrast, little is known about the expression of non-coding RNAs. Here we show that HCMV encoded miRNAs are expressed *de novo* during latent infection of primary myeloid cells. Furthermore, we demonstrate that miR-UL148D, one of the most highly expressed viral miRNAs during latent infection, directly targets the cellular receptor ACVR1B of the activin signalling axis. Consistent with this, we observed upregulation of ACVR1B expression during latent infection with a miR-UL148D deletion virus (ΔmiR-UL148D). Importantly, we observed that monocytes latently infected with ΔmiR-UL148D are more responsive to activin A stimulation, as demonstrated by their increased secretion of IL-6. Collectively, our data indicates miR-UL148D inhibits ACVR1B expression in latently infected cells to limit proinflammatory cytokine secretion, perhaps as an immune evasion strategy or to postpone cytokine-induced reactivation until conditions are more favourable. This is the first demonstration of an HCMV miRNA function during latency in primary myeloid cells, implicating that small RNA species may contribute significantly to latent infection.

Latent infection by human cytomegalovirus (HCMV) is maintained by a restricted viral transcription program where, in general, the majority of lytic cycle-associated genes are robustly repressed, resulting in a lack of infectious virus production^1^. In healthy seropositive individuals, one site of HCMV latency is in the cells of the myeloid lineage where latent genome is found in monocytes and their CD34^+^ haemopoietic progenitors^2^,^3^,^4^,^5^,^6^. Despite the fact that haemopoietic cell types derive from CD34^+^ progenitor cells, HCMV is only detectable in the myeloid cells of the peripheral blood and not lymphocytes^7^. This has led to the supposition that latent HCMV genome is carried selectively down the myeloid lineage. Although the mechanism by which this occurs is still unclear, it may involve the known increase in cellular GATA-2 during latency^8^,^9^ as GATA-2 is a well-established key regulator of hematopoiesis^10^.

It is also well established that lytic reactivation from latency can be triggered by differentiation of latently infected myeloid cells to dendritic cells or macrophages which ultimately results in the production of infectious virions. This has been reproducibly demonstrated in both experimental and natural latent infection where terminal differentiation of infected CD34^+^ cells or monocytes results in reactivation of virus from latency^2^,^3^,^11^,^12^,^13^,^14^,^15^,^16^.

During natural latency, a specific subset of viral genes (latency-associated genes) is expressed essentially in the absence of robust expression of lytic immediate early (IE) gene genes such as IE72 and IE86. This viral gene expression pattern can also be observed in experimental model of latency. For instance, HCMV infection of primary CD34^+^ cells or CD14^+^ monocytes, in defined medium to prevent any myeloid differentiation, results in the expression of latency-associated transcript UL138 in the virtual absence of the lytic IE72 transcript^2^,^17^. Whilst low levels of IE72 transcript have been observed using extremely sensitive RT-PCR in some studies^18^,^19^, the comparatively high levels of latency-associated transcripts compared to IE72 transcripts, which is in stark contrast to the relative super-abundance of IE72 transcription observed during lytic infection, is still consistent with a latent infection in undifferentiated myeloid cells. Importantly, a major criterion for latent infection is that no infectious virus is produced during the latent life cycle and in most, if not all, experimental latency models infectious virus is only detected after differentiation of the latently infected population^2^,^3^,^11^,^12^,^13^,^14^,^15^,^16^,^20^,^21^,^22^.

Whilst it is clear that the latent transcription programme of HCMV differs significantly from its lytic transcription programme, more recent analyses have suggested that latency-associated HCMV transcripts in primary myeloid cells may be more wide ranging than first thought^23^. In all cases, viral genes expressed during latent infection (by definition in the absence of robust IE gene expression) are also expressed during lytic infection. However, in contrast to latent infection, their expression during lytic infection is IE dependent^17^.

Besides viral protein coding genes, so-called non-coding RNAs (ncRNAs) are also expressed during HCMV lytic infection and these include viral microRNAs (miRNAs). There have been limited analyses of quality or quantity of expression of HCMV encoded microRNAs (miRNAs) during latent infection^24^,^25^,^26^, despite the fact that miRNA expression would be well-suited to the demands of the latent life cycle. As non-coding RNAs are capable of concurrently modulating the expression of multiple targets^23^,^26^,^27^,^28^ and altering gene expression at a global level, these RNAs are non-immunogenic molecules which, if expressed during latency, will not arouse detection of the latently infected cells by the immune system. This is a particularly important consideration during latency as the majority of the HCMV-encoded immune evasion genes are likely not expressed during this part of viral life cycle. To date, studies of HCMV miRNAs during latency have been restricted to analyses in the latent/quiescently infected THP-1 myelomonocytic cell line model^24^,^25^,^26^ as well as one recent analysis in monocytes of HCMV seropostive donors^26^. Although in this analysis^26^, there appeared to be little consistency between the small number of donors tested and the viral miRNAs detected.

HCMV is known to encode at least 20 miRNAs across its genome^25^,^29^,^30^ and, as important latency-associated roles have been identified for miRNAs encoded by other herpesviruses^31^,^32^,^33^,^34^,^35^, we postulated that these HCMV encoded miRNAs are also likely to contribute to HCMV latency. We, therefore, decided to determine if viral miRNAs are expressed in latently infected primary CD34^+^ progenitors and their derivative CD14^+^ monocytes and, if so, to identify potential latency-associated functions.

Here we show that, in experimentally latent primary CD34^+^ haemopoietic progenitor cells and primary monocytes, a number of HCMV encoded miRNAs are expressed to high levels *de novo.* We further show that one of these, miR-UL148D, targets cellular ACVR1B, a receptor of the activin signalling axis which has a number of functions in cellular processes but, importantly for HCMV biology, is also known to promote monocyte differentiation to dendritic cells (DCs). Whilst latent infection of monocytes, in itself, did not alter the low level of ACVR1B, levels of this receptor were upregulated during latent infection in monocytes in which miR-UL148D had been deleted. This suggests that latent infection increases the expression of ACVR1B, which is then returned to basal levels by miR-UL148D. Subsequent analysis of the ability of these cells to respond to activin A stimulation, by expression of cellular IL-6, showed that monocytes latently infected with wild-type virus secreted less IL-6 than monocytes latently infected with ΔmiR-UL148D. Taken together, these observations suggest that miR-UL148D is involved in fine-tuning of ACVR1B levels during latent infection which may restrict inflammatory cytokine production perhaps helping to postpone terminal differentiation of the monocytic cell until the conditions for full reactivation are optimal.

## Results

### HCMV encoded miRNAs are expressed during latent infection in CD34^+^ haemopoietic progenitor cells and CD14^+^ monocytes

We analysed expression of HCMV encoded miRNAs during latent infection by RT-qPCR, as detailed below. In these analyses, the primers used against miR-US4-1 were based on the originally defined miR-US4-1 sequence^36^ which was also used to characterise miR-US4-1 function by Kim *et al*.^37^. This sequence of miRUS4-1, however, differs from its later description by Stark *et al*.^29^ and Meshesha *et al*.^30^ at the 5′ end by 5 bases, though we do not know whether the primer in our assay is also able to detect this version of miR-US4-1.

Before miRNA expression levels during latent infection with HCMV were determined, we thought it essential to validate the bespoke qPCR detection system used in the study for their target HCMV encoded miRNAs. To confirm the presence of one single qPCR product arising from each primer set, the qPCR products were separated and visualised on agarose gels. Supplementary Fig. S1 shows amplification with all miRNA primer sets resulted predominantly in one single band, further confirming that the qPCR reactions amplify single gene products.

Secondly, as the analysis of relative miRNA levels was to be based on ΔΔCT values, it was important to ensure that target miRNAs had comparable amplification efficiency (within the range of 1.8–2.2)^38^. Therefore, all miRNA primers sets were assessed for amplification efficiency. All bar one primer set (against miR-UL69) were within the required range (Supplementary Table S1), thus the primer set against miR-UL69 was excluded from subsequent analysis.

Consequently, of the 20 experimentally verified HCMV encoded miRNAs listed in miRBase at the start of this analysis^39^, 18 were directly analysed. Due to experimental constraints, two HCMV encoded miRNAs were excluded. This included miR-UL69 (as detailed above) but also miR-UL70-3p, as it was not possible to generate a primer against the mature miRNA likely as a consequence of its G-rich nature. We also used cellular miR-16 as a reference miRNA as, in a screen for cellular miRNA expression changes caused by latent infection in CD34^+^ cells, miR-16 was deemed not to be altered by latent infection (<2 fold change)^8^. Moreover, miR-16 expression has also been shown not change with lytic infection in fibroblasts, during which all known latency-associated viral genes are also expressed^40^.

**Figure 1:**
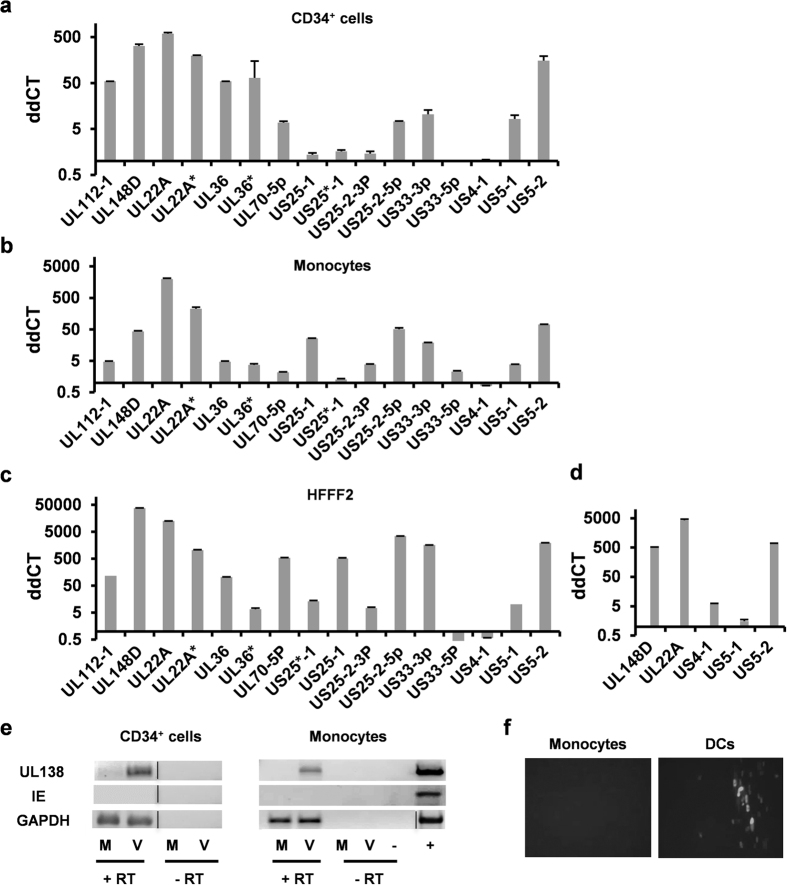
HCMV encoded miRNAs are expressed during experimental latency in CD34^+^ haemopoietic progenitor cells and monocytes. CD34^+^ haemopoietic progenitor cells **(a)**, monocytes **(b)** and HFFF2 fibroblasts **(c)** were either mock infected or infected with TB40 IE86-YFP before mature miRNA levels were assessed by RT-qPCR at 4 dpi. CD34^+^ cells and monocytes were infected at MOI 5 whilst HFFF2 were infected at MOI 2.5. **(d)** Monocytes were mock infected or infected with the parental WT TB40e virus from which the TB40 IE86-YFP virus was derived from at MOI 5, after which the level of viral miRNAs were analysed at 4 dpi. All data shown are representative of three independent experiments, and error bars marks standard deviation between technical replicates. The predominant guide strand and the minor passenger strand derived from the same miRNA duplex are denoted without and with an asterisk (*), respectively. The guide strand is thought to be responsible for most, if not all, mRNA downregulation by the miRNA. RNA from (**a,b**) were also analysed by RT-PCR against targets indicated in **(e)**. Monocytes infected with TB40 IE86-YFP were allowed to establish latency for 4 days before cells were or were not differentiated to mature dendritic cells. Cells were co-cultured with indicator fibroblasts for a further 5 days before the assessment of IE86 expression by fluorescence microscopy in **(f)**.

We analysed virally encoded miRNA expression in CD34^+^ (Fig. 1a) and CD14^+^ (Fig. 1b) cells at 4 days post infection (dpi) with TB40 IE86-YFP. Consistent with our previous analyses of experimentally latently infected CD34^+^ cells at this time point of infection^2^,^41^, these CD34^+^ cells showed a lack of IE mRNA and the presence of latency-associated UL138 transcript (Fig. 1e). Again, consistent with other previous analyses of CD14^+^ monocytes at this time point of experimental latent infection^19^, these CD14^+^ monocytes showed a lack of IE mRNA and the presence of latency-associated UL138 transcript (Fig. 1e) as well as the induction of IE protein expression only after their differentiation and maturation to interstitial dendritic cells (Fig. 1f). Similarly, no infectious virus was detectable in these experimentally latent monocyte cells (see below). These observations are all entirely consistent with previous analyses using these models which routinely show the lack of IE mRNA, the presence of latency-associated UL138 transcript as well as a lack of production of infectious virus^2^,^14^,^19^,^41^.

Figure 1 shows multiple virally encoded miRNAs were expressed during latent experimental infection of CD34^+^ (Fig. 1a) and CD14^+^ (Fig. 1b) cells at 4 dpi, representative raw data for which is shown in Supplementary Table S2. Furthermore, the expression profile of viral miRNAs during latent infection in the two different myeloid cell types were similar but did differ from the miRNA profile observed during lytic infection in fibroblasts (Fig. 1c). This suggested that there were latency and/or myelocytic cell specific expression profiles of HCMV encoded viral miRNAs.

The miRNA expression analyses described above were all performed with TB40 IE86-YFP^42^, a BAC-based recombinant HCMV known to be deleted for the viral US2-6 region and therefore missing miR-US4-1. Consistent with this, miR-US4-1 was not detected in cells infected with this BAC-derived TB40 IE86-YFP (Fig. 1a–c). Similarly, as miR-US5-1 and miR-US5-2 are proximal to the US2-6 region (they are located at the intergenic region between US6 and US7), it was also possible that their expression might have been compromised by use of the BAC-based virus. Consequently, we analysed the expression of miR-US4-1 and re-analysed the expression of miR-US5-1 and miR-US5-2 miRNAs after latent infection in monocytes with a wild-type (WT) TB40e virus. As Fig. 1d shows, the expression levels of miR-US5-1 and miR-US5-2 during latent infection with WT TB40e were similar to that of TB40 IE86-YFP; miR-US5-2 is highly expressed whilst miR-US5-1 is expressed to a lower level. The expression of miR-US4-1, which is not present in TB40 IE86-YFP, was detected at low levels in cells latently infected with WT TB40e virus and this was at levels similar to miR-US5-1 expression during latent infection.

**Figure 2:**
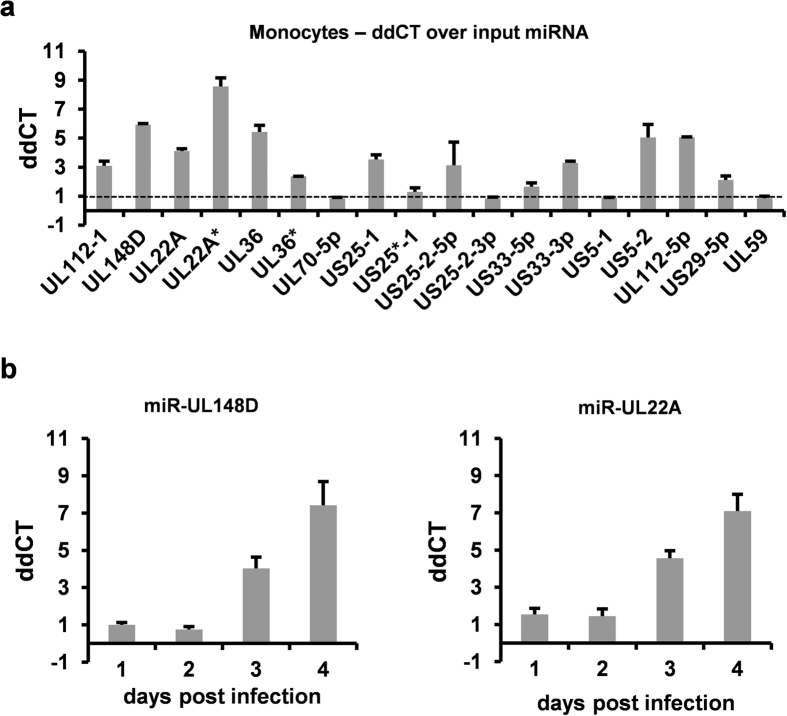
HCMV encoded miRNAs are *de novo* expressed during latent infection in monocytes. **(a)** Monocytes were infected with TB40 IE86-YFP or equivalent UV-inactivated virus at MOI 5 before miRNA levels were analysed 4 dpi. The miRNAs present in UV-inactivated virus treated control indicated the input miRNA level, and this served as the baseline from which miRNA expression was measured. **(b)** Monocytes were infected with TB40 GFP WT or equivalent UV-inactivated virus at MOI 5 after which miRNA levels were analysed at time points indicated. Expression levels over input are shown.

HCMV is known to incorporate both viral and cellular RNA into virions^43^,^44^,^45^. Consequently, we were minded that the experimental latent infection used for the studies could be confounded by incoming miRNAs indiscriminately incorporated into virions. Therefore, we also examined levels of viral miRNAs in cells infected with UV-inactivated virus to ensure that viral miRNAs identified in latently infected CD34^+^ cells and CD14^+^ monocytes were derived from *de novo* synthesised RNAs during latent infection and not input virion RNA. To do this, CD14^+^ monocytes were infected with TB40 IE86-YFP or UV-inactivated virus and RT-qPCRs for viral miRNAs were carried out, as before. In this analysis, which was subsequent to the analysis in Fig. 1, we also included, in addition, two more recently identified miRNAs (miR-UL59 and miR-US22). Using input RNA from UV inactivated virus as reference level, Fig. 2a shows that most viral miRNAs could be detected in latently infected cells at levels well above levels detected in cells infected with UV-inactivated virus controls at 4 dpi, confirming that the viral miRNAs identified after latent infection of CD34^+^ cells and monocytes were expressed *de novo* during latent infection. Furthermore, whilst the use of input RNA as reference resulted in substantially lower expression levels of viral miRNAs compared to Fig. 1, suggesting miRNAs contained in virions do contribute to signals observed immediately upon infection, *de novo* expression of a number of viral miRNAs clearly occurred. Importantly, the use of UV-inactivated virus controls also allowed us to identify that some of the viral miRNAs, apparently expressed at very low levels during latency, were likely a result of virion-associated input miRNAs. On this basis, miR-UL70-5p, miR-US25^*^-1, miR-US25-2-3p, miR-UL59 and miR-US5-1 appear not to be expressed to any appreciable levels during experimental latency, at least in CD14^+^ monocytes (Fig. 2a). This is in contrast to lytically infected cells in which miR-UL70-5p, miR-US25*-1, miR-US25-2-3p and miR-US5-1 are clearly expressed (30,36,46,47,48,49 and see Fig. 1c) and indirectly confirms the data in Fig. 1 which showed that the CD34^+^ and monocytes used for these analyses were, indeed, latently infected. Similarly, it has been reported that in infected THP-1 cells, miR-US25-2-3p and miR-US5-1 are only expressed if the THP-1 cells are differentiated^24^. We also examined the time course of expression of two of the highly expressed miRNAs, miR-UL148D and miR-UL22A. This analysis showed that expression of these viral miRNAs did not become established until 3 dpi and accumulated further at 4 dpi (Fig. 2b), supporting the view that expression of these viral miRNAs was associated with latent infection, rather than e.g. as a consequence of an initial burst of lytic infection.

Collectively, these data suggest that a number of HCMV encoded miRNAs are expressed to high levels during experimental latent infection in CD34^+^ haemopoietic progenitor cells and monocytes; one such miRNA was miR-UL148D, which was routinely observed to be robustly expressed during latent infection.

### MiR-UL148D in isolation targets ACVR1B

Two cellular targets of miR-UL148D have been identified previously. One is RANTES (CCL5), which has been confirmed experimentally during HCMV lytic infection in fibroblasts^37^, whilst the other, IEX-1, has been shown to be suppressed by miR-UL148D in transfection assays in HEK293 cells^50^.

To investigate other possible cellular functions modulated by miR-UL148D expression during latent infection, we employed two computer prediction algorithms, Reptar and RNAhybrid^51^,^52^, which have both been used previously to successfully identify cellular targets for HCMV miRNAs^53^,^54^. Of a number of predicted targets identified by each of the two computer algorithms, one cellular target was identified by both algorithms and, importantly, the sites identified by each algorithm were identical - both algorithms showed identical target sites in the 3′ UTR of the activin A receptor, ACVR1B. ACVR1B is a part of the activin signalling cascade which is activated by activin A, a member of the TGF-β ligand superfamily. In immune cells, activin A activates the pathway by inducing the formation of type I (ACVR1B)/type II receptor heterodimers, which results in downstream signalling through a number of intermediaries including Smad proteins, which TGF-β also signals through^55^. The activin signalling axis is ubiquitously used by many different immune cells (amongst multiple other cells types) to regulate a diverse range of biological processes, though the functional outcome is broad-ranging and may either pro-inflammatory or anti-inflammatory depending on the cell type and context^56^. In the myeloid lineage, activin A has been observed to govern migration, pro- and anti-inflammatory cytokine production as well as differentiation and maturation in a cell-type specific manner (Table 1)^57^,^58^,^59^,^60^,^61^,^62^,^63^. Because of the roles of activin A in inflammation and differentiation, processes that might be key targets for latent infection, we believed there was a strong possibility that ACVR1B was a *bona fide* target of miR-UL148D during latency and pursued a functional analysis of this miRNA target in the context of latent infection.

**Figure 3:**
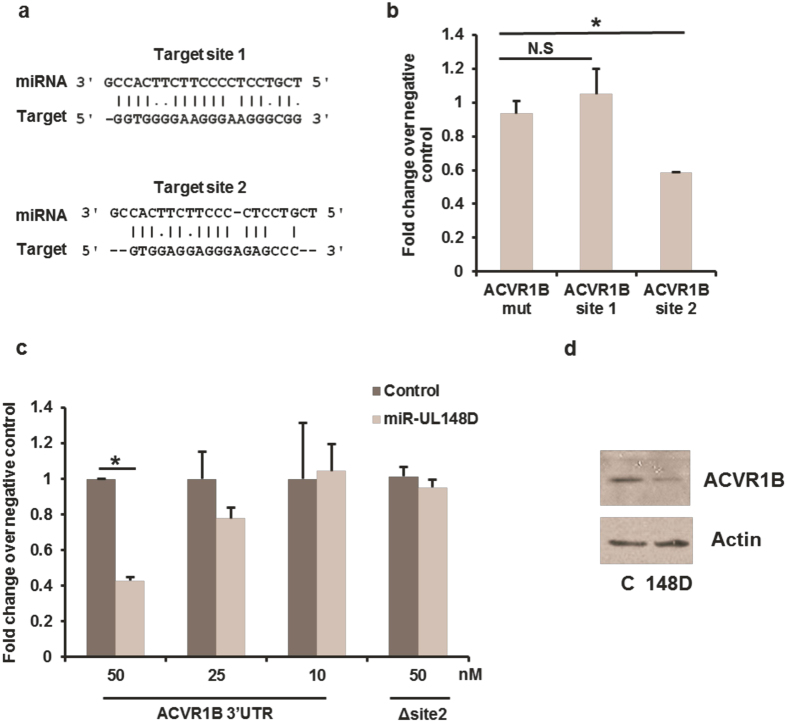
ACVR1B 3′ UTR is directly repressed by miR-UL148D at one of two predicted target sites. The computer algorithms Reptar and RNAhybrid predict miR-UL148D to target the ACVR1B 3′ UTR at two separate non-canonical sites; both are shown in **(a)**. **(b)** 293T cells were transfected with a negative control siRNA or miR-UL148D mimic together with a construct containing either an irrelevant sequence (ACVR1B mut), ACVR1B target site 1 or 2 was inserted in the 3′ UTR of the luciferase reporter gene. At 24 hours post transfection, luciferase activity was determined, and fold change in luciferase activity in the miR-UL148D transfected cell compared to negative control is displayed. **(c)** 293T cells were transfected with a negative control siRNA or miR-UL148D mimic at concentrations indicated together with a luciferase reporter vector containing the entire ACVR1B 3′UTR with (ACVR1B 3′UTR) or without (Δ site 2). Luciferase activity was determined at 24 hours post transfection and data is shown as described in (**b**). Significant differences were determined using *t*-test and marked by (*p < 0.05) whilst N.S. denotes not significant differences. **(d)** KG-1 cells were transfected with negative control siRNA (C) or miR-UL148D mimic (148D), after which ACVR1B levels were assessed by western blot at 48 hours post transfection. The autoradiograph shown has been cropped with no further manipulation.

As the computer prediction algorithms identified two identical sites in the 3′ UTR of ACVR1B as potential targets of miR-UL148D (Fig. 3a), we interrogated these predicted miR-UL148D target sites in ACVR1B individually by constructing luciferase expression vectors containing each of the predicted miR-UL148D binding sites shown in Fig. 3a. Although it is conventional to demonstrate the targeting of a putative miRNA to its binding site by mutating one or two bases within the putative seed sequence, the predicted sites for miR-UL148D binding in ACVR1B were non-canonical. Consequently, we reasoned that mutating the seed sequence alone may not be sufficient to prevent target site binding by the miRNA. Therefore, we chose to replace the predicted binding sites in the ACVR1B 3′ untranslated region with an irrelevant sequence of equal length to act as a negative control vector (ACVR1B mut). Each of the vectors were co-transfected into HEK93T cells together with either a negative control siRNA or a miR-UL148D mimic. Whilst luciferase activity of the target site 1 construct or the irrelevant control vector (ACVR1B mut) was not affected by miR-UL148D, luciferase activity of the target site 2 construct was substantially repressed (Fig. 3b). Similarly, co-transfection of a vector containing the entire ACVR1B 3′UTR with miR-UL148D resulted in reduction of luciferase activity which was abolished when the target site 2 was deleted (Fig. 3c). A dose-dependent inhibition of ACVR1B 3′ UTR by miR-UL148D was also observable (Fig. 3c).

We next asked whether delivery of a miR-UL148D mimic, in isolation, into myeloid cells affected ACVR1B expression. Figure 3d shows that transfection of KG1 cells, a CD34^+^ myelomonocytic cell line which is relatively easily transfectable, with a miR-UL148D mimic resulted in a profound decrease in expression of ACVR1B which was not observed with a control irrelevant miRNA.

### MiR-UL148D targets ACVR1B during latent infection

In order to confirm that this effect of miR-UL148D on ACVR1B also occurred specifically in the context of latent infection, we next analysed expression of ACVR1B in monocytes latently infected with WT virus or with a virus lacking miR-UL148D. To do this, a miR-UL148D (ΔmiR-UL148D) virus deletion was made by disrupting the hairpin structure of pre-miR-UL148D by synonymous mutations such that maturation of the pre-miRNA no longer occurred; the lack of miR-UL148D expression in monocytes latently infected with this ΔmiR-UL148D virus was confirmed (Supplementary Fig. S2). In addition, the viruses have the GFP reporter gene driven by the SV40 promoter, thus allowing infected cells within a population to be identified.

**Figure 4:**
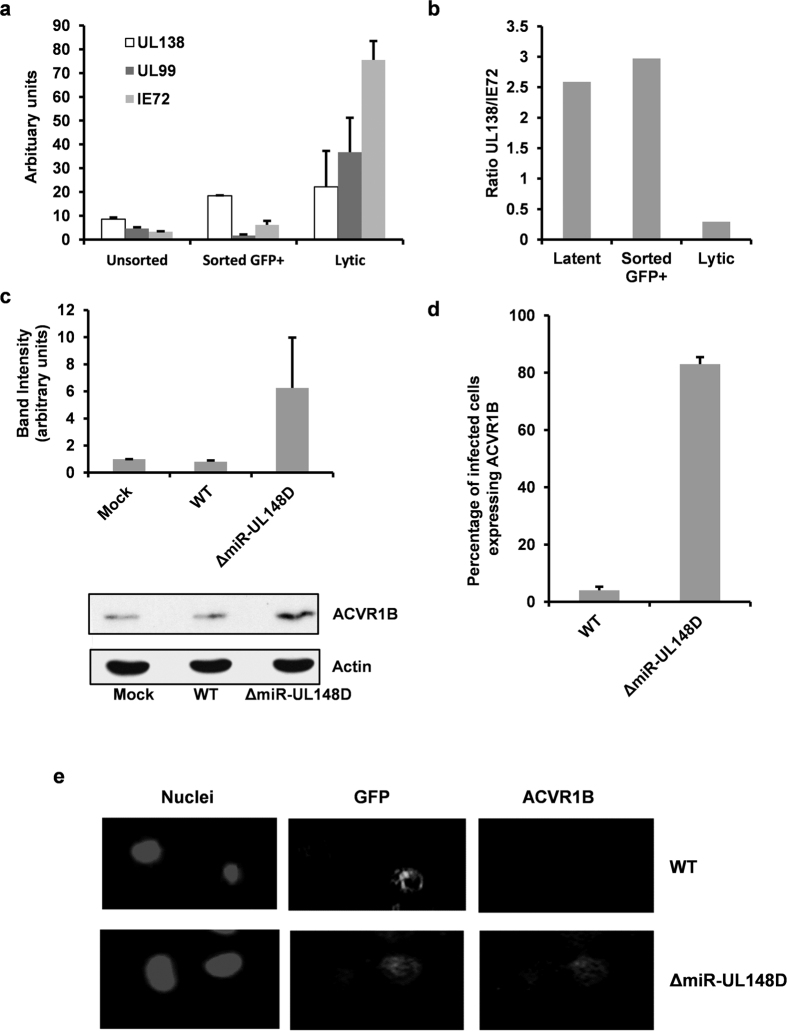
ACVR1B is downregulated by miR-UL148D during latent infection in monocytes. Monoytes were infected with TB40 GFP WT virus at MOI 5 whilst HFFF cells were infected at MOI 1. At 4 dpi, monocytes were either unsorted or sorted for GFP expression before monocytes and HFFF cells were then immediately harvested. Viral transcript level were analysed by RT-qPCR in **(a)** and the ratio of UL138:IE72 transcripts are shown in **(b)**. **(c)** Monocytes were infected with TB40 GFP WT or ΔmiR-UL148D at MOI 5, after which GFP expressing latently infected cells were sorted at 4 dpi, and were immediately harvested. Protein levels of ACVR1B and loading control actin were examined by western blot. After analysing densitometry by ImageJ (National Institute of Health), ACVR1B levels were normalised to actin, which is displayed alongside a representative blot. Each band of the blot has been cropped from the same autoradiograph with no further manipulation. The error bars denote the standard deviation of three independent experiments. In addition, ACVR1B expression in unsorted cells at 4 dpi was also analysed by immunofluorescence. The staining is enumerated in **(b)**, where 5 random fields of view of 100 cells per condition were counted for two independent experiments, and an example is shown in **(c)**.

First, we investigated whether infection of monocytes with this virus resulted in GFP expressing cells that are latently infected; for instance, it could be argued that the GFP expressing cells are undergoing e.g. spontaneous reactivation. However, both the unsorted population as well as cells sorted for GFP expression contained latency-associated UL138 mRNA at a high level whilst lytic transcripts IE72 and UL99 were near-absent (Fig. 4a). Importantly, the ratio of UL138:IE72 mRNA was equally high in both unsorted and sorted cells (Fig. 4b), suggesting GFP expressing cells are, indeed, latently infected and not, for instance, undergoing spontaneous reactivation. The ability of WT virus or ΔmiR-UL148D to affect ACVR1B during latent infection was then analysed. Monocytes latently infected with either virus were sorted from bystander uninfected cells on the basis of GFP expression and relative levels of ACVR1B expression on these cells was quantified by western blot analysis. Figure 4 shows that, at 4 dpi, latent infection with WT virus had little effect on the already relatively low levels of ACVR1B expression on monocytes. However, ACVR1B expression was induced up to six-fold in cells latently infected with ΔmiR-UL148D virus (Fig. 4c). Of note, although ACVR1B expression was observed to be reproducibly higher in ΔmiR-UL148D virus infected cells compared to WT infected cells in independent experiments, this did not reach statistical significance (as determined by *t*-test, *p* < 0.05). However, we also analysed the effects on ACVR1B expression using indirect immunofluorescence in unsorted latently infected cells at 4 dpi. Figure 4d,e also show that monocytes latently infected with miR-UL148D virus had substantially higher levels of expression of ACVR1B.

### The presence of miR-UL148D during latency suppresses activin A induced proinflammatory cytokine induction in latently infected monocytes

On the basis that miR-UL148D was expressed at high levels during latent infection and clearly targeted ACVR1B, we next asked whether miR-UL148D played any role in cellular functions important for latent carriage and/or reactivation in primary monocytes models of latency. Firstly, we confirmed that ΔmiR-UL148D virus was not defective for lytic infection in fibroblasts (Supplementary Fig. S3), as any defect in lytic cycle progression could be mistaken for inefficient reactivation.

**Figure 5:**
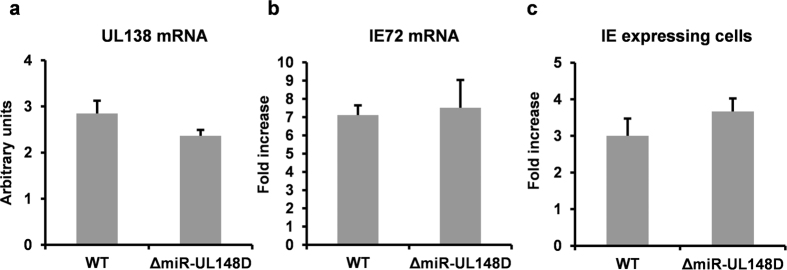
The deletion of miR-UL148D does not affect viral gene expression during HCMV latency or reactivation. Monocytes were infected with TB40 GFP WT or ΔmiR-UL148D at MOI 5 before UL138 transcript levels at 10 dpi were analysed by RT-qPCR **(a)**. Viral transcript levels were normalised to the mRNA level of housekeeping gene GAPDH. To assess reactivation, infected monocytes were differentiated to M2-like macrophages by 400 nM prednisolone^19^ from 3 dpi onwards for 7 days after which induced IE72 mRNA transcript **(b)** and IE expression **(c)** at 10 dpi were analysed by RT-qPCR and immunofluorescence, respectively. Expression is shown as fold increase over cells not stimulated to reactivate. Error bars denote the standard deviation of technical repeats. Data shown is representative of four independent experiments.

**Figure 6:**
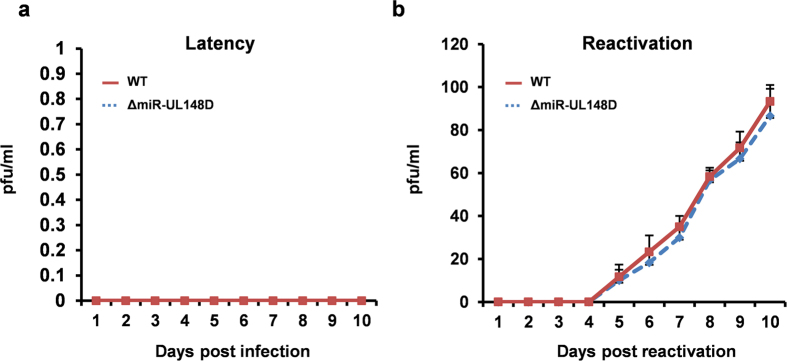
WT and Δ148D viruses establish latency and reactivate infectious virus equivalently. Monocytes were infected with either WT or ΔmiR-UL148D and latency established for 10 days before differentiation into dendritic cells with GM-CSF/IL-4 and LPS. After this time cells were co-cultured with HFFFs. Supernatants were collected on each day during latent infection in **(a)**, and after reactivation in **(b)**, which were tested on HFFFs for the presence of virus. The graphs represent triplicate samples and the virus production is measured in pfu/ml.

We next analysed the effect of miR-UL148D deletion on virus latency and reactivation. Infection of monocytes with WT and ΔmiR-UL148D, at equivalent MOIs, resulted in equivalent levels of UL138 mRNA, suggesting latency establishment was not affected by deletion of miR-UL148D (Fig. 5a). Consistent with this, co-culture of infected monocytes with HFFF fibroblasts from 1 to 10 dpi also did not yield infectious virus (Fig. 6a). Furthermore, the deletion of miR-UL148D did not affect the ability of virus to reactivate IE gene expression (Fig. 5b,c) or infectious virus upon terminal differentiation of monocytes (which were detected by co-culture with HFFF cells in Fig. 6b). Of note, we used two different standard methods for reactivation; one by prednisolone induced differentiation of monocytes to M2-like macrophages^19^ (as shown in in Fig. 5) or by differentiation to mature dendritic cells using IL-4/GM-CSF and LPS^3^ (as shown in Fig. 6). Deletion of miR-UL148D did not impair reactivation in either reactivation model. Collectively, these data suggest that miR-UL148D, and hence levels of ACVR1B, do not directly affect latency establishment or terminal differentiation-induced reactivation in experimentally latent cultures. However, miR-UL148D clearly targets ACVR1B, both in isolation and during experimental latent infection, suggesting that perturbation of other myeloid-specific functions of ACVR1B is important during latency. In support of this rationale, we observed that miR-UL148D is also expressed by at least one other clinical isolate, Titan^64^, during latent infection (Supplementary Fig. S4), and that miR-UL148D is fully conserved in all HCMV genomes that have been directly sequenced from clinical material to date (Andrew Davison, personal communication).

One common function of activin A, through ACVR1B engagement, is the modulation of secreted cytokines^57^,^58^,^59^,^65^. For instance, in monocytes and monocytic cell lines, activin A has been reported to have a proinflammatory effect by upregulating IL-6, IL-1α and TNF-α in resting monocytes^57^,^58^. In contrast, in cells that have been differentiated to DCs, activin A acts as a negative regulator by suppressing inflammatory cytokine expression^65^, including IL-6, and this is known to suppress HCMV reactivation of IE gene expression as IL-6 promotes efficient reactivation of HCMV from immature interstitial-like DCs^66^. Otherwise, the effect of activin A on herpesviral infection is largely undocumented.

**Figure 7:**
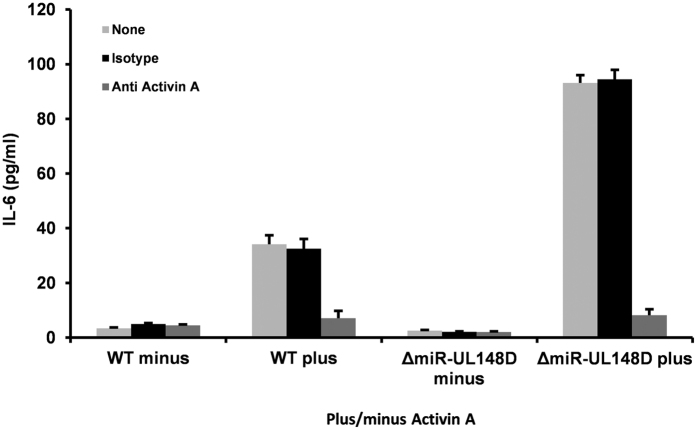
Targeting of ACVR1B by miR-UL148D during latency decreases induction of IL-6 in response to activin A. Monocytes were infected with either TB40 GFP WT or ΔmiR-UL148D for 3 days before the cells were treated with (plus) or without (minus) activin A for a further 24 hours in the presence or absence of neutralising antibodies to activin A or the specific isotype matched control as indicated. At this 4 day latency time-point, the supernatants were assessed for levels of IL-6 by ELISA. Data shown represents triplicate samples.

Consequently, we reasoned that careful control of levels of pro-inflammatory IL-6 expression may be required during latent infection in myeloid cells; firstly, to prevent the untimely appearance of cytokines which may give pro-reactivation signals and secondly, once myeloid differentiation has initiated, to help stimulate reactivation. Clearly, a mechanism by which this could occur would be through latency-associated regulation of ACVR1B expression. To test this, we infected monocytes with WT or ΔmiR-UL148D virus for 3 days and, after a further 24 h in the presence or absence of activin A, we analysed the induction of IL-6 in these latently infected cells (confirmed as latent at this time point of 4 dpi by the lack of detectable infectious virus production, see Fig. 6a). It is already well established that activin A increases expression of IL-6 in CD14^+^ monocytes^57^. However, what effect activin A has on latently infected monocytes, especially in the context of the presence or absence of miR-UL148D, is not known. Figure 7 shows that activin A treatment of cells latently infected with WT HCMV also show a discernible increase in expression of IL-6 but that this is substantially increased in cells latently infected with ΔmiR-UL148D virus. On the basis that GFP expression shows an average of 19.74% (range 14.83–25.79%, n = 6; Supplementary Fig. S5) of the cell population is latently infected, we think it likely that the increase in activin A induced IL-6 expression in WT latently infected cells is due to by-stander uninfected cells responding to activin A through their unperturbed ACVR1B and that the increase in IL-6 expression in cells latently infected with ΔmiR-UL148D results from an increase in response to activin A as a result of increase in their levels of ACVR1B expression. Consistent with this, antibody blocking of activin A abolished activin A stimulated IL-6 secretion from WT and ΔmiR-UL148D infected cells (Fig. 7). This data further supports our model that activin A acts through its cognate receptor to upregulate IL-6, and that the higher expression of ACVR1B in ΔmiR-UL148D infected cells sensitizes the cells to activin A stimulated IL-6 secretion.

## Discussion

As viral miRNAs have been documented to facilitate the establishment and maintenance of latency in other herpesviruses such as EBV, HSV and MHV68^31^,^32^,^33^,^34^,^35^, it is also likely that HCMV exploits this regulatory mechanism to optimise latent infection. Here, we have shown, for the first time, the *de novo* expression of viral miRNAs during HCMV latency in experimental models of both CD34^+^ haemopoietic progenitor cells and CD14^+^ monocytes.

It is worth saying that, on the basis that approximately 20% of monocytes in our analyses were latently infected, the relative levels of miRNA expression between HFFFs (100% infected) and monocytes suggest that miRNA expression during latency may be at levels not too dissimilar to levels obtained during lytic infection.

Perhaps not unexpectedly, we observed that some of the apparent low level expression of viral miRNAs during latent infection resulted from RNA contamination of virion preparations. Once corrected for, there were only a handful of viral miRNAs which appeared to be expressed at high levels during latent infection whilst others, although expressed at high levels during lytic infection, were poorly expressed during latency.

In contrast to α and γ herpesvirus, in which miRNAs are often encoded in clusters and derived from the same pri-miRNA^31^,^67^, little is currently known about how HCMV encoded miRNAs are regulated either during lytic or latent infection. However, the observation that HCMV encoded miRNAs are dispersed throughout the viral genome, may help explain the ability to differentially regulate viral miRNA expression during the latent and lytic life cycles.

One of the most abundantly expressed viral miRNA we observed during latent infection was miR-UL148D; this was expressed in both latently infected monocytes and CD34^+^ cells. Although deletion of this miRNA from the viral genome showed it was not critical for the ability of the virus to establish latency or undergo reactivation in monocytes, it was clearly involved in targeting the cellular receptor ACVR1B, both in isolation and during experimental latency. Furthermore, our data suggests that this targeting of ACVR1B by miR-UL148D downregulates the secretion of IL-6 in response to activin A in these latently infected monocytes, arguing that the role of miR-UL148D during latency is at the level of modulating monocyte functional interactions to optimise latent carriage rather than direct effects on latent genome carriage and reactivation. For instance, previous reports suggest that naturally occurring regulatory T cells are likely to help create an immunosuppressive microenvironment in the vicinity of latently infected cells^68^. As it is well established that IL-6 can block the activity of regulatory T cells, whilst enhancing the expansion and cytotoxic activity of cytokine-induced killer cells^69^,^70^,^71^, secretion of IL-6 by latently infected cells could antagonise such an immunosuppressive environment by inhibiting the activity of local regulatory T cells. Furthermore, IL-6 downregulation would also likely prevent IL-6 mediated activation of NK cells, a cell type that is important in the control of HCMV lytic infection^72^,^73^,^74^. Previous work by Hook *et al*.^75^ has demonstrated miR-US5-1, miR-US5-2 and miR-UL112-1 inhibits TNF-α and IL-6 release during lytic infection by downregulating multiple components of the endocytic pathway. As we also observed that these miRNAs are expressed during latency, they may act cooperatively with miR-UL148D to limit proinflammatory cytokine release during this latent phase of the virus life cycle. It is also possible that miR-UL148D may additionally target other proinflammatory cytokines directly or indirectly to further reinforce any immune evasive activity, and these avenues await further investigation potentially in tractable animal models of HCMV latency in which e.g. T cell responses to latent HCMV, as has been observed *in vivo*^68^, have been documented.

The effect of IL-6 on latent infection and reactivation in myeloid cells has been analysed^66^. Whilst IL-6 does not stimulate reactivation of virus from latently infected monocytes, it does stimulate efficient HCMV reactivation from immature interstitial-like dendritic cells^66^. Therefore, the latency-associated reduction in the expression of ACVR1B, mediated by miR-UL148D, and the subsequent reduction in the ability of latently infected cells to respond to activin A with an increase in secretion of IL-6, could also affect the ability of any monocyte derived immature DCs to reactivate virus in suboptimal conditions. This suggests that the downregulation of IL-6 in e.g. latently infected myeloid cells could be a part of a concerted effort by the virus to prevent untimely reactivation events. It is worth pointing out that exogenous IL-6 is known to reactivate HCMV from immature DCs^66^. A pertinent question is, then, why the increases in IL-6 observed during experimental latent infection of monocytes with the miR-UL148D deletion virus does not, in itself, induce reactivation of latently infected monocytes. Firstly, IL-6 cannot induce reactivation of virus from latently infected monocytes, only from differentiated immature monocyte-derived DCs^66^ and, secondly, the levels of IL-6 induced upon latent infection of monocytes with miR-UL148D deletion virus (which is in the picogram range in our analyses) is far below the levels of exogenous IL-6 (in the nanogram range) required to reactivate virus from immature monocyte-derived DCs^66^.

Of note, IL-6 downregulation appears to be a phenomenon shared by at least one other herpesvirus. The transfection of HSV-1 miR-H6 into cornea epithelial cells decreased their secretion of IL-6 through a currently unknown mechanism^76^ and suggests that the downregulation of IL-6 may be important to herpesvirus infections, in general.

Our observation that HCMV encoded miRNAs are expressed during latent infection has opened up a number of potential avenues of investigation into how HCMV miRNAs may contribute to latency. With each miRNA likely regulating tens to hundreds of cellular or viral targets^27^, expression of viral miRNAs during HCMV is one way to orchestrate a plethora of cellular functions to optimise the cells for latent carriage and reactivation.

## Methods

### Cell culture

G-CSF mobilized primary CD34^+^ haemopoietic progenitor cells from healthy donors with average purity of 98.9% (range 97.8 to 99.8% n = 3) were commercially sourced (Stemcell Technologies). Primary monocytes were isolated from venous blood by CD14^+^ MACS microbeads positive selection (Miltenyi Biotec) as the manufacturer instructs. Briefly, peripheral blood mononuclear cells were extracted from venous blood by Lymphoprep density gradient centrifugation (Axis-Shield), as previously described^77^. PBMCs were then incubated with CD14 direct microbeads before application of a magnetized LS column, and subsequently eluting the bound cells after washing the column. The purity of isolated cells were analysed by flow cytometry, which showed 98.1% cells expressed CD14 (range 97.4–98.9%, n = 5). Both primary CD34^+^ cells and monocytes were cultured in X-Vivo-15 (Lonza) supplemented with 2.5 mM L-glutamine (GE healthcare).

HFFF2 fibroblasts were cultured in EMEM-10, whilst KG-1 cells were maintained in RPMI-10.

### Experimental latent infection and reactivation

WT TB40e and TB40 IE86-YFP have previously been described^42^,^78^. TB40 GFP WT and TB40 GFP ΔmiR-UL148D were constructed by Eain Murphy’s laboratory, where the GFP reporter is under the SV40 promoter^14^,^79^.

Latent infections in CD34^+^ cells or monocytes were achieved as previously described^2^. Briefly, cells were incubated with HCMV at the MOI 5 at 37 °C with intermittent agitation for 3 hours, after which cells were washed once before incubation with fresh media. Analysis of GFP expression at 2–4 dpi of cells incubated with TB40 GFP virus showed infections were achieved in 14.83–25.79% cells. CD34^+^ cells or monocytes were left for 3–4 days after incubation with virus to achieve latency, after which monocytes were differentiated to mature dendritic cells or M2- like macrophages for reactivation, as previously detailed^19^,^66^. No overt signs of differential cell death were observed between mock-infected and infected culture by light microscopy during any time point of the experiments.

For comparison of reactivation efficiency between virus strains, the cells containing reactivating virus were co-cultured following with HFFF cells and supernatant removed at daily intervals and stored at −80 °C until ready for further analysis. After 10 days all supernatants were defrosted and used to inoculate fresh HFFF cells. These were then left for a further 7 days to form plaques. After this time cells were fixed and stained for IE and pfu/ml calculated.

### RT-PCR and RT-qPCR

For RT-PCR, RNA was harvested using TRIzol reagent (Life technologies) following the manufacturer’s instructions, after which they were then digested using RQ1 RNase-free DNAse system (Promega). Reverse transcriptions were performed using the Reverse Transcription System (Promega) in accordance with the manufacturer’s instructions. PCR reactions were performed using BioMIx Red PCR reaction mix (Bioline) for GAPDH (forward – GAGTCAACGGATTTGGTCGT, reverse – TTGATTTTGGAGGGATCTCG), IE exon 2/3 (forward – GGACCCTGATAATCCTGACG, reverse – ATCTTTCTCGGGGTTCTCGT) and UL138 (forward – TGCGCATGTTTCTGAGCTAC, reverse – ACGGGTTTCAACAGATCGAC). PCRs were run for 35 to 45 cycles (95 °C for 45 s, 55 °C for 45 s, 72 °C for 1 min) with a long denaturing step before the cycles (95 °C for 10 minutes) and a long elongation step at the end (72 °C for 10 minutes). For RT-qPCR, RNA was isolated using RNeasy mini kit (Qiagen) before one-step RT-qPCR was performed using Quantitect Virus kit (Qiagen) as previously described^80^.

### RT-qPCR of miRNAs

Total RNA was extracted using miRNeasy Mini Kit (Qiagen) according to manufacturer’s instructions and eluted in RNAse-free water. Two-step RT-qPCRs were then performed using the miSCRIPT PCR system (Qiagen) as manufacturer instructs using primer assays against mature HCMV miRNAs. All qPCR reactions were performed in duplicate with no template controls and no reverse transcriptase controls. The levels of miRNAs were analysed using ΔΔCT method with the cellular miR-16 as reference.

### Luciferase reporter assays

Luciferase reporter constructs were created using the pMIR-REPORT Vector system (Life technologies) as detailed by the manufacturer using the Spe1 and either HindIII or MluI restriction sites. The sequence inserted was GGTGGGGAAGGGAAGGGCGG for ACVR1B site 1, and TGGAGGAGGGAGAGCCCA for ACVR1B site 2. The irrelevant sequence used for to create the ACVR1B mut control was GTTTTGTTTTGGTTTGGTTGTG, which was predicted not to be targeted by miR-UL148D by RNAhybrid. To insert the entire ACVR1B 3′ UTR (GenBank accession number NM_004302) into the vector, the sequence was amplified from human genomic DNA (Bioline) using forward primer TTTTTACTAGTAAGATCTAACTGCTCCCTCT and reverse primer TTTTACGCGTTTAGAACATCCACCCCATTC using the Expand High Fidelity PCR System (Roche) as the manufacturer instructs. Once the ACVR1B 3′ UTR luciferase construct had been obtained, it was used as the template to create the ACVR1B 3′ UTR Δ target site 2 construct through PCR deletion mutagenesis, using the forward primer CAAGCTTTGGCAGAGAACT and reverse primer TGTCCCTGCCCTCAGG.

Luciferase reporter assays in Fig. 3b were performed by co-transfecting 293T cells with a combination of 240 nM miR-UL148D mimic or Allstar negative control siRNA (both from Qiagen), 50 ng luciferase reporter plasmid and 450 ng carrier plasmid pBluescript (Agilent Technologies) using 1–2 μl Jetprime transfection reagent (Polyplus transfection). Luciferase activity was then analysed at 24 hours post transfection as previously described^8^. Experiments in Fig. 3c were performed identically except for changes in the concentrations of siRNA/mimic (indicated in the Figure), the luciferase reporter plasmids (0.5 ng), and the luciferase activity was assessed using Luciferase Assay System (Promega, cat no. E1500).

### KG-1 cells transfection

Cells were transfected by electroporation using the Amaxa Nucleofector kit R (Lonza) as the manufacturer instructs. Briefly, 2 × 10^6^ cells were transfected with 300 μM of either Allstar negative control siRNA or miR-UL148D mimic (both manufactured by Qiagen). Protein levels were analysed by western blot at 48 hours post transfection.

### Sodium dodecyl sulphate-polyacrylamide gel electrophoresis (SDS-PAGE) and western blotting

Samples were resolved by running on a 10% SDS-PAGE gel using Mini-PROTEAN Tetra Cell (Bio-Rad, Hercules, California, USA) before being transferred to Hybond nitrocellulose membranes (GE Healthcare). Proteins were probed for by incubation with the primary antibody against either ACVR1B (Abcam, cat no. ab109300/ab133478) or Actin (Abcam, cat no. ab8227). This was followed by incubation with horse radish peroxide (HRP)-linked secondary antibody (Santa Cruz Biotechnology, cat no. sc-2004/sc-2963) at room temperature for 1 hour. Proteins were then detected through using chemiluminescence (ECL plus, GE Healthcare) that were exposed to X-ray films (Super RX, Fujifilm) for autoradiographic analysis. Densitometry analysis was performed using ImageJ (National Institute of Health), as previously described^81^.

### Immunofluorescence

Cells were fixed using 70% Ethanol and then washed in PBS before treating with a 1 in 500 dilution in PBS antibody to ACVR1B (Abcam, cat no. ab109300) or an isotype control. Cells were stained for 1 hour prior to 3 × 5 minute washes in PBS and staining for 1 hour with nuclear stain 1 μg/ml Hoechst 33342 (Sigma-Aldrich) and 2 μg/ml goat anti-rabbit Alexa Fluor 594 (Molecular Probes; Thermo Fisher scientific). Following 3 × 5 minute washes in PBS, cells were visualised by fluorescence microscopy.

### IL-6 secretion

Cells were treated without or with 100 nM Activin A (Peprotech) for 24 hours in the presence or absence of 0.2 μg of neutralising antibody to Activin A (R&D systems) or 0.2 μg of the relevant isotype control antibody (R&D systems) before secreted IL-6 levels were analysed by ELISA (R&D Systems).

### Ethics Statement

Ethical permission for this project was granted by the Cambridgeshire 2 Research Ethics Committee (REC reference 97/092). Written informed consent was obtained from all of the volunteers included in this study prior to providing blood samples for the preparation of primary CD14^+^ monocytes. Primary CD34^+^ haematopoietic progenitors were from normal hematopoietic stem cell transplant donors after stem cell mobilization in accordance with the Declaration of Helsinki (Lonza).

## Additional Information

**How to cite this article**: Lau, B. *et al*. The Expression of Human Cytomegalovirus MicroRNA MiR-UL148D during Latent Infection in Primary Myeloid Cells Inhibits Activin A-triggered Secretion of IL-6. *Sci. Rep.* **6**, 31205; doi: 10.1038/srep31205 (2016).

## Supplementary Material

Supplementary Information

## Figures and Tables

**Table 1:** Functions of myeloid cells regulated by activin A.

Cell type	Experimental system	Effects	Reference
Monocytes	Monocytes (PBMCs), THP-1 and U-937 cells	Activin A ↑ IL-6, IL-1α and TNF-α, (resting), but ↓ IL-1β and ↑ sIL-1Ra in activated monocytes	57,58
	Monocytes, Intradermal injection of activin A in human skin explants cultures	Activin A promoted LC differentiation	60
Macrophages	THP-1 cells differentiated to macrophage phenotype	Activin A ↓ TNF-α and IL-8 induced by LPS	82
	Monocyte-derived macrophages	Skews polarize macrophage towards a proinflammatory (M1) phenotype	61,62
Dendritic Cells	Co-cultures of mo-DCs and autologous T cells	Blocking of CD40L-induced activin A ↑ cIL-10, TNF-α, IL-6 and IL-12p70, MCP-1, RANTES, IL-8 and IP-10 and ↑ T-cell proliferation	65
	Immature mo-DCs and peripheral DCs	Activin A ↑ CXCL12, CXCL14, MMP2, MMP3 and MMP9 production	59
	Immature mo-DCs	Activin A induces directional migration through ACVR1A and ACVR1B	59
	Mo-DCs cultures	Activin A and inhibin A ↓ maturation of mo-DCs	63

Activin A regulates a variety of functions in different myeloid cell types. LC indicates Langerhans dendritic cell whilst mo-DCs indicates monocyte-derived dendritic cells. Table is adapted from^83^.
