# Corrigendum: The Expression of Human Cytomegalovirus MicroRNA MiR-UL148D during Latent Infection in Primary Myeloid Cells Inhibits Activin A-triggered Secretion of IL-6

**DOI:** 10.1038/srep33771

**Published:** 2016-09-26

**Authors:** Betty Lau, Emma Poole, Benjamin Krishna, Immaculada Montanuy, Mark R. Wills, Eain Murphy, John Sinclair

Scientific Reports 6: Article number: 31205; 10.1038/srep31205 published online: 08 05 2016; updated: 09 26 2016.

The original version of this Article contained an error in the spelling of the author Immaculada Montanuy which was incorrectly given as Immaculada Sellart.

In addition, the Author Contributions Statement contained errors.

B.L., E.P., B.K. and I.S. performed the experiments and analysed the data. E.M. provided the viruses. B.L., E.P. and J.S. wrote the manuscript whilst all authors discussed the results and contributed to the manuscript.

now reads:

B.L., E.P., B.K., I.M. and M.W. performed the experiments and analysed the data. E.M. provided the viruses. B.L., E.P. and J.S. wrote the manuscript whilst all authors discussed the results and contributed to the manuscript.

These errors have now been corrected in the PDF and HTML versions of the Article.

