# Supplementary material for: The Expression of Human Cytomegalovirus MicroRNA MiR-UL148D during Latent Infection in Primary Myeloid Cells Inhibits Activin A-triggered Secretion of IL-6
Source: Sci Rep. 2016 Aug 5;6:31205. doi: 10.1038/srep31205 (PMC4974560; doi:10.1038/srep31205)
Supplement: Supplementary Information [file srep31205-s1.doc]

Supplementary Information for

**The Expression of Human Cytomegalovirus MicroRNA MiR-UL148D during Latent Infection in Primary Myeloid Cells Inhibits Activin A-triggered Secretion of IL-6**

Betty Lau, Emma Poole, Benjamin Krishna, Immaculada Sellart, Mark R. Wills, Eain Murphy,and­ John Sinclair

**Figure S1. MiRNA qPCR reactions amplify a single major PCR product.** RNA from latently infected monocytes was reverse transcribed before qPCR reactions were performed using the miScript system (Qiagen). The products of the qPCR were then resolved in 2% agarose gel. M indicates marker Hyperladder 50bp (Bioline).

**Figure S2. ΔmiR-UL148D virus does not express miR-UL148.** Monocytes were mock, WT or ΔmiR-UL148D virus infected at MOI 5 before mature miRNA levels were analysed by qPCR at 4 dpi. Error bar denotes standard deviation of technical replicates.

**Figure S3. ΔmiR-UL148D virus does not have a growth defect.** HFFF2 fibroblasts were infected with TB40 GFP WT (solid red line) or ΔmiR-UL148D (blue dashed line) at MOI 1 **(a)** and 0.01 **(b)**. Cell-free infectious virus present in the supernatant were analysed by TCID50 on days post infection indicated. Data shown is representative of technical replicates.

**Figure S4. HCMV clinical isolate Titan expresses viral miRNAs during latency.** Monocytes were infected with Titan or equivalent UV-treated virus at MOI 5 before mature miRNA levels were analysed at 4 dpi. The expression over UV treated controls are shown, whilst error bars denote standard deviation between technical repeats.

**Figure S5. An average of 19.74% of monocytes infected with TB40 GFP WT or TB40 GFP ΔmiR-UL148D express GFP at 3 dpi**. Monocytes were infected at MOI 5 before GFP expression was analysed by flow cytometry. Plots shown are representative of three independent experiments.

**Table S1.** **Amplification efficiencies of miRNA specific primers.** cDNA from infected HFFF2 were five-fold serial diluted to generate a standard curve. QPCR was then performed in duplicate using primers against specific HCMV encoded miRNAs as listed. The slope, the correlation coefficient (R2), and the amplification efficiency of the miRNA primers were extrapolated from the standard curve.

**Table S2. CT values from miRNA qPCRs of latently infected CD34+ cells and monocytes.** Samples were run in duplicate and results from a representative experiment are shown.
